# Exploring the Current Situation and Developing Strategies for Behavior Change to Improve Antibiotic Use in West Africa: Protocol for a Multidisciplinary Interventional Research Project

**DOI:** 10.2196/66424

**Published:** 2025-07-25

**Authors:** Maresa Neuerer, Carine Baxerres, Denise Dekker, Blandine Bila, Daniel Arhinful, Leslie Mawuli Aglanu, Charity Wiafe Akenten, Boubacar Coulibaly, Ali Sié, John Humphrey Amuasi, Aurélia Souares

**Affiliations:** 1 Heidelberg Institute of Global Health Heidelberg University Heidelberg Germany; 2 Institut de Recherche pour le Développement Laboratoire Population Environnement Développement Aix-Marseille Université Marseille France; 3 Bernhard Nocht Institute for Tropical Medicine Hamburg Germany; 4 Département Biomedical/Santé Publique Institut de Recherche en Sciences de la Santé Ouagadougou Burkina Faso; 5 Noguchi Memorial Institute for Medical Research University of Ghana Accra Ghana; 6 Kumasi Centre for Collaborative Research in Tropical Medicine Kumasi Ghana; 7 University Medical Centre Groningen University of Groningen Groningen The Netherlands; 8 Centre de Recherche en Santé de Nouna Nouna Burkina Faso; 9 School of Public Health Kwame Nkrumah University of Science and Technology Kumasi Ghana; 10 German Center for Infection Research Heidelberg Germany

**Keywords:** antimicrobial resistance, One Health, design thinking, multidisciplinary, intervention, sub-Sahara Africa, mixed methods

## Abstract

**Background:**

Antimicrobial resistance (AMR) is recognized as a global concern, with particularly severe consequences in low- and middle-income countries. Although it may occur naturally, it is also an anthropogenic problem linked to the irrational use of antibiotics in humans and animal husbandry and the use of pesticides in plant agriculture. Generally, data on AMR and evidence of effective and feasible multifaceted interventions are limited in many African countries.

**Objective:**

This study aims at (1) assessing baseline data on AMR pathogens in Burkina Faso and Ghana; (2) understanding perceptions and quantifying use of antibiotics among health care workers, communities, and livestock farmers; and (3) defining and refining an AMR intervention using a design thinking approach.

**Methods:**

This multidisciplinary study will be conducted in two rural districts and will consist of two phases. First, baseline data will be collected on AMR pathogens along dominant food production chains. extended-spectrum beta-lactamase (ESBL)–producing Escherichia coli and Klebsiella pneumoniae will be selected as indicator mechanisms for AMR because of their high occurrence among animals and humans. The perception and understanding of AMR and antibiotic use among different stakeholders and community members will be assessed using epidemiologic and socio-anthropological methods. Qualitative methods will include participant observations, in-depth and key informant interviews, and focus group discussions. The quantitative part will consist of the development of an inventory of circulating antibiotics and a household survey. Second, key informant and in-depth interviews will be conducted with One Health stakeholders in preparation for the intervention development. Subsequently, multidisciplinary “design teams” will develop ideas for an intervention on AMR using a design thinking approach.

**Results:**

Data collection started in April 2022. The analysis of microbiological, anthropological, and socio-epidemiological data is ongoing in both countries. The intervention development has been initiated in Ghana but has not started yet in Burkina Faso. All results are planned to be submitted to peer-reviewed journals by December 2025. First, manuscripts will be published for each discipline. Afterward, the results of the 3 disciplines will be combined in multidisciplinary papers, and a publication of the evaluation of the intervention will follow.

**Conclusions:**

Owing to the multifactorial nature of AMR, different perspectives need to be considered to develop a holistic context-based intervention that is tailored to local needs. This study stands out in its combination of different disciplinary and epistemological perspectives following the One Health paradigm and taking a design thinking approach to develop an intervention. Thereby, collaboration across disciplines and social levels and a participatory bottom-up approach will be promoted to achieve a common understanding of problems and needs and to develop an accepted and efficacious intervention. The national AMR networks and policymakers will be continuously involved in the project.

**International Registered Report Identifier (IRRID):**

DERR1-10.2196/66424

## Introduction

### Overview

Antimicrobial resistance (AMR) is rising to dangerously high levels in all parts of the world and presents a threat to public health, food security, and (economic) development [1]. Currently, AMR is presented as being responsible for 700,000 deaths per year and could result in 10 million annual deaths per year by 2050 if no action is taken [1]. As new resistance mechanisms are emerging and spreading globally, the ability to treat common infectious diseases and enable chemotherapies, operations, and many other activities is threatened [2,3]. It is acknowledged as an urgent global problem that concerns every country irrespective of its level of income and development, but effects are hypothesized to be even more significant in low- and middle-income countries (LMIC) [1,4]. In social sciences, however, experts highlight how AMR can be exploited for political ends [5], the invisibility of several causes of AMR [6], and significant scientific knowledge gaps on this subject [7]. Legido-Quigley et al [8] described AMR as a “wicked problem,” as it encompasses complex networks of different stakeholders with incompatible interests.

AMR is spread via diverse agents including humans, animals, alimentation, soil, and water. Although resistance can emerge naturally through evolutionary processes [9,10], it can also occur through anthropogenic pressure resulting from an overreliance on antibiotics. Infections in humans and animals caused by extended-spectrum beta-lactamase (ESBL)–producing *Escherichia coli* and *Klebsiella pneumoniae* are increasingly resistant to most antibiotics globally due to their high diversity and rapid mutation [11,12]. The evaluation of these two gram-negative bacteria could serve as an important indicator for the prevalence of AMR in local settings.

It is critical to recognize how human practices interact with natural microbial ecology in compounding AMR risks. Within the human health sector, the reliance on broad-spectrum antibiotics, especially in the absence of diagnostics, has been identified as a major contributor to the emergence of AMR [13]. The widespread use of antibiotics in livestock exceeds direct human consumption and is driven by economic imperatives to maximize production, prevent disease, and enhance growth [7,14,15]. As most of these antibiotics are not fully metabolized but released into the environment as waste products, misguided antibiotic use has a concurrent ecological impact by affecting wild bacteria populations and promoting further antibiotic resistance development [16]. This highlights the ecological consequences of antibiotic misuse and the entangled nature of environmental, agricultural, and health systems. Additionally, the soil contains microorganisms with naturally occurring genes that can facilitate the development of AMR in the natural environment [3,7].

The relevance of this research project is multifaceted: First, the availability of information on AMR in Africa is limited. This stems from weak laboratory and surveillance systems and processes, where only a small fraction of facilities conducts bacteriological testing and the microbiological data that do exist are often of poor quality [4,17,18]. Similarly, more data on antibiotic consumption and access to health care in animal and human health are needed [19]. In addition, a One Health approach should be implemented in future studies to develop efficient interventions on AMR and improve AMR surveillance and control in Africa [19,20]. Second, the rates of AMR are higher in certain LMIC, while individual antibiotic consumption is lower in comparison with high-income countries, indicating that other factors are influencing the prevalence of AMR [9]. Third, the effects of AMR on various societal sectors, including health and economic consequences, risk being more significant in African countries due to predominant livelihoods and living conditions [21,22]. In recent years, the use of antibiotics in LMIC has increased substantially both for humans and animals because of economic growth and changes in diet [23]. At the same time, antibiotic policies concerning manufacture, dispensation, and prescription in many LMIC are often missing or lacking regulatory enforcement due to socioeconomic challenges, large population size, and challenged health care systems [24]. Fourth, the current body of literature “has created little clarity about what interventions are best suited to achieve AMR goals across contexts, cultures and health systems” [25]. The need to act on AMR at a global level is well-known and agreed upon; however, an implementation gap of action and interventions can be observed [26]. Evidence of effective and feasible multifaceted interventions in sub-Saharan African countries is limited, and studies show that challenges for implementation of interventions are numerous. Nevertheless, several initiatives at international and local levels have demonstrated that effective interventions are feasible in LMIC and that contextualization is essential [27].

### Study Objectives

This paper presents the study protocol for a multidisciplinary research project on AMR in 2 districts of Burkina Faso and Ghana using a One Health perspective and design thinking approach. The study has the following aims: (1) assessing baseline data on AMR pathogens along the food chain in the Kossi Province, Burkina Faso, and the Asante Akim North District, Ghana; (2) understanding and quantifying perceptions and use of antibiotics among health care workers, communities, and livestock farmers as well as defining patterns of access and utilization of antibiotics; and (3) defining and refining an intervention aimed at reducing the impact of AMR and improving the way this issue is addressed using a stakeholder-driven approach.

## Methods

### Study Setting

The research project will be conducted in Burkina Faso and Ghana, both located in the West African subregion. The research site in Burkina Faso is the rural Kossi Province, located in the northwest of the country and corresponding to the Nouna Health District. The population is mostly living in the semi-urban town Nouna, representing the administrative center of the province [28]. Nouna Health District has a population size of about 320,232 inhabitants and a surface area of 7464 km^2^ [28]. The main economic activities are farming and animal breeding [29]. A Health and Demographic Surveillance System has been established in the province and is managed by the Centre de Recherche en santé de Nouna (CRSN; Nouna Health Research Center). Burkina Faso has a national human and animal surveillance system for AMR consisting of 15 designated laboratories since 2018 and is enrolled in the Global Antimicrobial Resistance and Use Surveillance System (GLASS) of the World Health Organization (WHO) but is not yet reporting data to the system [30]. Burkina Faso’s National Action Plan (NAP) was published in 2018 but has not been implemented by the government yet [30].

The research site in Ghana is in the semi-urban and rural Asante Akim North District, which is in the eastern part of the Ashanti Region. The district has a size of 1218 km^2^ and approximately 85,788 inhabitants [31]. Approximately 72% of the district’s work force is employed in the agrarian sector [32]. Multiple health institutions are serving in the district and are constituted by private sector actors, religious missions, and public sector actors [32]. Ghana is enrolled in the WHO GLASS. However, there is no precise nationwide surveillance system on antimicrobial use and AMR prevalence, and no data were contributed in 2020 and 2021 [33-36]. The establishment of a nationwide AMR surveillance system is outlined in the NAP but has yet to be implemented [36,37]. Ghana is considered a leading country on the African continent for handling AMR due to its work and approach including the establishment, in 2011, of the Ghana National Policy Platform on AMR, which consists of various stakeholders from diverse institutions and sectors, thereby integrating the One Health approach [36]. Moreover, the NAP on AMR (2017-2021) and a policy document on antimicrobial use and resistance were officially launched in April 2018 in the country [36,38]. However, policy and project implementation is lagging due to poor coordination of the different stakeholders, insufficient financial resources and infrastructure, and enforcement of regulations [36].

Ghana and Burkina Faso are neighboring countries but different in many respects, such as the socioeconomic level, the structure of the health system, the presence of industries, and national security. Therefore, it would be of interest to contrast the information collected between the two study sites and to see the influence of these factors on AMR and what this could mean for possible interventions. In addition, through the development of a south-south collaboration between the two countries, knowledge can be shared, and synergy effects can be used for a holistic approach to AMR.

### Study Design

#### Overview

**Figure 1:**
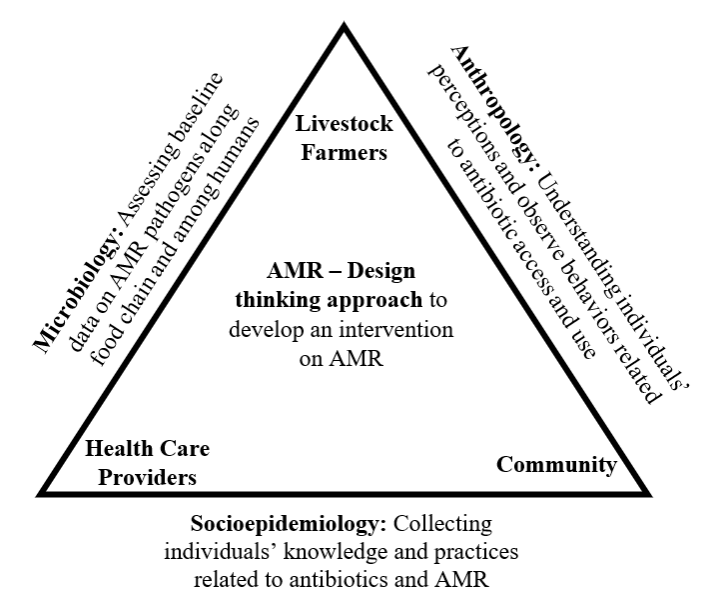
Research design: triangulation between 3 disciplines to develop an intervention using a design thinking approach. AMR: antimicrobial resistance.

This research project consists of 2 phases (Figure 1). Phase 1 consists of multidisciplinary research on AMR, looking at it from different perspectives, namely, microbiology, anthropology, and socioepidemiology. This will consist of baseline data collection on AMR pathogens (ie, microbiology [1.1.]) We will explore perceptions and use of antibiotics among health care workers, communities, subsistence and semi-intensive livestock farmers, and institutional actors (ie, anthropology [1.2.] and socioepidemiology [1.3.]). Thereby, we are following an exploratory sequential mixed methods design by initially using qualitative methods to then develop quantitative tools in the next step. In the second phase, we will use a design thinking approach to develop a targeted intervention in collaboration with different stakeholders to improve the way of addressing the issue of AMR (ie, interventional research component [2.]). The identified stakeholders will be involved from the beginning of the project, with their opinions and perceptions of the problem and suggested solutions prioritized in the design thinking process.

#### Phase 1.1: Microbiology

First, we will assess baseline data on AMR pathogens along the dominant food chains: poultry in Ghana and poultry and beef in Burkina Faso. ESBL-producing *E. coli* and *K. pneumoniae* will be selected as the indicator mechanisms due to their high prevalence among animals and humans. Farms will be included in the sample if their poultry or cattle are sent to the local abattoir and sold in sampled local markets and retail shops. Due to local practices, the AMR prevalence will also be tested among free-range, so-called backyard, chickens in the villages.

A total of 1500 fecal samples will be collected from poultry, along with 500 meat samples from markets, 200 from retail shops, and 100 from abattoirs. During each sampling visit, individual fecal droppings from broilers (on poultry farms) or cattle (on farms) will be collected in sterile containers. These samples will then be placed in a transport box maintained at 2 °C to 4 °C to ensure proper preservation. In markets, retail shops, and the abattoir, meat samples of approximately 1 gram each will be collected into a sterile plastic bag and transported on ice to the Kumasi Centre for Collaborative Research in Tropical Medicine laboratory in Kumasi. In a biosafety cabinet, the samples will be transferred into sterile disposable petri dishes, and the meat will be diced into smaller pieces with a sterile surgical blade and transferred into Brain Heart Infusion (Oxoid) broth. After incubation at 37 °C for 18 hours to 24 hours in normal atmosphere, the broth will be plated on Chromagar supplemented with ESBL-selective antibiotics (CHROMagar) and incubated at 37 °C for 18 hours to 24 hours. Presumptive *E.* *coli* colonies and *K. pneumoniae* colonies will be subcultured onto Columbia blood agar and further tested using biochemical methods. Antibiotic sensitivity testing will be performed using the Kirby-Bauer method, and ESBL confirmation will be conducted using the double-disk diffusion method. ESBL-producing bacteria under investigation will be subjected to whole genome sequencing to describe phylogenies as well as virulence and AMR. Data will be entered into a REDCap database, and bacteriological data will be analyzed using STATA (StataCorp). The number and percentage of samples along with descriptive statistics (ie, median, IQR, minimum, and maximum) will be reported for continuous non-normally distributed data. Categorical variables will have the number and percentage of patients reported. Proportions of positive test results will be calculated as percentages of positive results among all valid tests, with exact binomial 95% CIs.

#### Phase 1.2: Anthropology

Ethnographical studies, including daily immersions, participant observations, free discussions, and semistructured interviews, will be used. For the qualitative investigations in human health, a research assistant will be living in a community area of the rural town where the research will take place (Nouna in Burkina Faso, Agogo in Ghana) for a total of 12 months. He or she will become part of the community and, during an exploratory phase, will observe and talk to as many people as he or she meets during their daily life (neighbors; the people he or she meets when shopping, eating, and investigating places where medicines are distributed and care is provided). During the 12 months, he or she will travel to a rural locality around where he or she will live for 2 months and become integrated and that he or she will explore in the same way as previously in the town. Subsequently, he or she will conduct semistructured interviews in 15 households in the urban community and in 15 households in the rural locality (if possible, with the mother and the father). These households will have at least 1 child younger than 10 years and will be chosen to represent different socioeconomic statuses (“affluent” households, “intermediate” households, “deprived” households).

For the qualitative investigations in poultry farms, ethnography will be performed in approximately one-half of the active farms in the rural town in each country. We will choose the farms according to their operating time (from before 2000 until now) and the number of animals raised (500 to 8000 chickens).

Antibiotic use by individuals and farmers and waste management practices at the household, farm, and health care system levels will be investigated. In addition to antibiotic use, the overall living and working conditions, constraints experienced, and factors influencing health-seeking behaviors for both humans and animals will be studied. We will also explore practices of professional actors in biomedicine and veterinary medicine in addition to developing an exhaustive inventory of antibiotics in circulation. Semistructured interview guides used within the anthropology work package for the ethnographies conducted on farms and villages can be found in Multimedia Appendix 1 and Multimedia Appendix 2, respectively, and the observation guide for households in the villages for human health and animal health is shown in Multimedia Appendix 3. All other observation guides can be accessed from the corresponding author upon request. All the data will be analyzed using thematic sorting in NVivo 14, identifying redundant themes and subthemes that are relevant to understanding the social realities being studied.

#### Phase 1.3: Socioepidemiology Quantitative

A household survey about the perception of AMR and the use of antibiotics will be implemented at the community level. In Ghana, a double-stage random sampling method will be used, while in Burkina Faso, simple randomization will be used due to the availability of a Health and Demographic Surveillance System. The sample will include approximately 1000 households per country. The questionnaire will be designed using the Multi-Country Public Awareness Survey from the WHO and previous literature. It will consist of 2 sections: one for the head of the household, covering socioeconomic status, demographics, and antibiotic use in livestock and another focusing on knowledge, attitudes, and practices (KAP) related to antibiotics and AMR awareness. Participants belonging to a household will be selected randomly by the interviewer. Data collection will be conducted using tablets by two separate teams in Ghana and Burkina Faso, both of whom will undergo a 5-day training before the start of fieldwork activities.

Data will be analyzed using STATA 17. Descriptive analysis will be conducted to summarize sample characteristics using frequencies and percentages for categorical data and means with standard deviations for linear data. Socioeconomic status will be calculated using principal component analysis and classified into quintiles. KAP will be assessed by assigning points to correct responses; respondents will then be categorized into groups based on their scores. Regression analysis will be conducted for both countries to explore associations. Collinearity will be assessed to ensure the reliability of the regression models.

We will look at the circulation of antibiotics in the formal sector in the two districts analyzing the register of the district pharmacy for a period of 2 years. Subsequently, we will investigate the overall quantity of antibiotics according to antibiotic classes and the AWaRe classification. Results will be reported to the general population of the district.

#### Phase 1.3: Socioepidemiology Qualitative

A qualitative survey focusing on prescription patterns and perceptions of AMR will be conducted among health care providers of primary and secondary health care facilities, including Community-based Health Planning and Service compounds, health centers, policlinics, and a district hospital, using in-depth interviews and focus group discussions. Sampling will be done using a stratified purposeful sampling strategy aiming for maximum variation within the 3 strata, namely nurses, physician assistants, and medical doctors. The semistructured interview guide that will be used for this can be found in Multimedia Appendix 4. The semistructured guide developed for the focus group discussions can be accessed from the corresponding author on request. Content analysis will be applied to data coding and analysis with the COM-B (capacity, opportunity, motivation, behavior) model as the underlying framework [39].

#### Phase 2: Interventional Research Component

**Figure 2:**
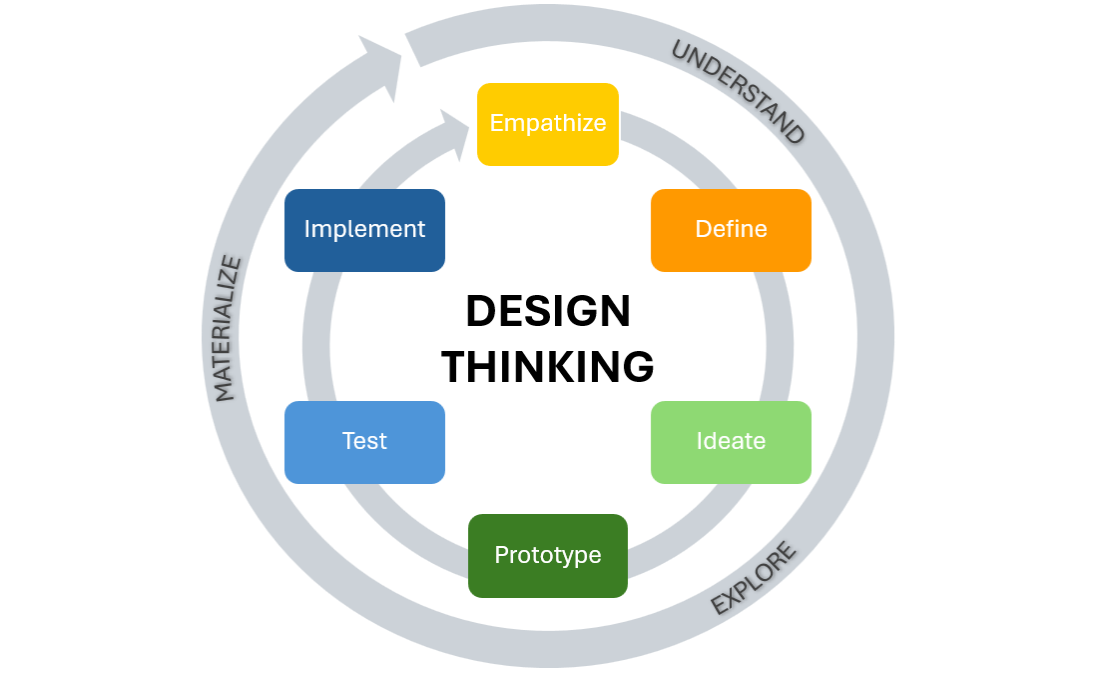
Description of the design thinking approach.

Using the design thinking approach (Figure 2), a targeted intervention to improve the way of addressing the issue of AMR will be developed in collaboration with different stakeholders in the second phase [40]. Within a system of overlapping nonsequential and iterative spaces of understanding, exploring, and materializing, the following steps will be repeated over time: empathize, define, ideate, prototype, test, and implement [41].

Building on the results of the previous study phase and in preparation for the workshops, key informant interviews will be conducted with institutional actors, and in-depth interviews will be conducted with community members and farmers to understand the perception and understanding of AMR among different One Health stakeholders. Institutional actors will be selected via purposeful sampling and will be eligible if involved in a One Health sector, namely animal, environmental, and human health. Community members and farmers will be sampled using a customer-exit strategy at points of sales of antibiotics. The semistructured interview guides used for interviews with farmers and community members can be found in Multimedia Appendix 5 and Multimedia Appendix 6, respectively. Interview guides for institutional actors have been jointly developed by the anthropology and socioepidemiology team and will slightly differ according to the sector of the interview partners. An example of the interview guide for the human health sector can be found in Multimedia Appendix 7. The two remaining interview guides for institutional stakeholders in the environmental and human health sector can be accessed from the corresponding author upon request. All interviews will be transcribed verbatim in French or English. Subsequently, we will code transcripts through a mixed approach of inductive and deductive coding using thematic analysis.

The design thinking approach follows a human-centered and emerging design and is a collaborative and iterative process aiming to produce relevant results according to the needs and local situation [42,43]. Therefore, initial ideation workshops in both study sites will combine the research results collected in the first phase of the project. Research results will be discussed by workshop participants before collecting ideas on possible interventions on AMR through participatory methods. Therefore, multidisciplinary and collaborative “design teams” will be developed and include the research team, policymakers from the involved countries, and members of nongovernmental organizations working on AMR as well as health care workers, veterinary professionals, livestock farmers, and community members. Discussions will be recorded and transcribed, followed by qualitative data analysis to summarize the ideas generated for an intervention on AMR. Based on these results, the design teams will meet again in each study site to develop the prototype of an intervention. Although the outcome of the design teams’ work will depend on this participatory process, we assume that the intervention could possibly be targeting the community level due to the variety of stakeholders involved in the intervention development. Generally, interventions at the community level, such as education interventions, have been shown to result in positive changes in knowledge and attitudes; however, there is a lack of evidence on the effect of improved knowledge on behavior change [44].

The intervention will be implemented in Kossi Province and Asante Akim North District and evaluated using a mixed methods triangulation approach combining questionnaires and interviews with stakeholders at different levels to evaluate the process and outcome of the intervention. This will allow refinement and modification of the intervention according to the local needs. Eventually, dissemination meetings will be organized, and policy briefs will be written to ensure wide access to the results by policymakers and scientists in the countries of interest and abroad. In addition, continuous contact with policymakers and the national AMR networks in Burkina Faso and Ghana from the beginning of the research process has the potential to increase the acceptability and scaling of the developed intervention.

### Ethical Considerations

Ethical approval was obtained from the ethical committee of Heidelberg University Hospital (S-826/2021), National Ethical Committee in Burkina Faso (number 2021-12-270), Nouna Health Research Centre ethical committee (number 2021-018-MS/SG/INSP/CRSN/CIE), Kwame Nkrumah University of Science and Technology Committee on Human Research Publication and Ethics (CHRPE/AP/580/21), and Ghana Health Service Ethics Review Committee (GHS-ERC 004/11/21).

Informed consent and the ability of participants to opt out of the study at any point will be provided together with an information sheet on the project. Additionally, all data will be de-identified to ensure privacy and confidentiality. All recordings will be deleted once transcription is completed and checked. Participants of the workshops in phase 2 will receive beverages and a meal during the workshops and compensation for their attendance once the workshop has ended, as this presents a day-long commitment. Otherwise, no compensation will be provided for study participants.

## Results

The study received funding in May 2021. Ethical approvals were granted from the various committees between November 2021 and April 2022. Renewal of the ethical approval is requested and received annually from the Kwame Nkrumah University of Science and Technology Committee on Human Research Publication and Ethics.

Data collection started in April 2022 and was completed in November 2023. For the quantitative socioepidemiology part, we conducted a cross-sectional survey among 1114 participants in Ghana and 1011 participants in Burkina Faso. These numbers were based on prior sample size calculation and reflect completed data collection as of November 2023. Qualitative components in the anthropology work package included daily immersions, participant observations, free discussions, and semistructured interviews, and semistructured interviews and focus group discussions in the socioepidemiology work package. Given the exploratory nature of these methods, participants were recruited progressively across multiple target groups, with data collection continuing until saturation was reached.

The analysis of the microbiological, anthropological, and socioepidemiological data is ongoing in both countries. The intervention development has been initiated, and the first prototype meeting took place in Ghana in July 2024. The pretesting of the intervention in Ghana is expected to be completed by August 2025. The process of intervention development has not started yet in Burkina Faso, as we are experiencing a delay due to the political situation. All results are planned to be published in peer-reviewed journals before December 2025. Initially, the 3 disciplines involved in the research will analyze their data separately while exchanging information on a regular basis. This will also allow us to put the different results into perspective. For example, to combine microbiological and anthropological results, parasite resistance rates on a specific farm will be considered alongside observations of antibiotic use on the same farm. This could include an assessment of whether resistances described by microbiologists in the geographical contexts studied can be understood based on the descriptions of perceptions and uses of medicines produced by anthropologists in the same context. The congruence or noncongruence of the overall analysis between the disciplines will then be discussed and problematized. Eventually, without strictly comparing our results on each of the questions addressed, we will propose scientific articles providing an overall interpretation of AMR, cross-referenced between microbiology and anthropology and between microbiology, socioepidemiology, and anthropology. Results will not be strictly compared for each question addressed but will be embedded in a holistic analysis of AMR in the specific study contexts.

First, manuscripts will be published for each discipline. Afterward, the results of the 3 disciplines will be combined in multidisciplinary papers, and a publication of the evaluation of the intervention will follow.

## Discussion

### Overview

Research has shown that “magic bullet” approaches to AMR focusing on highly technological and biomedical approaches such as surveillance and the development of new antibiotics will not be sufficient to combat the global and multifactorial problem of AMR [13,45]. Even though we currently do not have much data on the prevailing situation in the regions we will be studying, we know that the irrational use of antibiotics in humans and animals significantly contributes to AMR [3,10,26]. Policies and underlying infrastructure of antibiotics, perceptions, understanding, and knowledge of stakeholders and community members need to be understood to develop sustainable and effective solutions while considering their social and economic capital as well as behavioral, ethical, political, and cultural factors [4,15,26,45]. Therefore, the multifactorial nature of AMR, the diverse reasons and drivers underlying the use of antibiotics, and the local contexts and constraints need to be considered and integrated into all efforts.

### Phase 1

The first phase of the research project will add to the field of One Health by examining the issue of AMR from microbiology, anthropology, and socioepidemiology perspectives. Therefore, this study will address the challenge of disciplinary silos through close multidisciplinary collaboration. It stands out in the combination of different disciplinary and epistemological perspectives through multilateral collaboration between the Burkinabe, Ghanaian, German, and French research institutions. Microbiological data will contribute to a clear picture of prevalent resistance in animals and improve the biomedical understanding of AMR in the study sites. Generally, both AMR surveillance and data on AMR are limited in western sub-Saharan Africa [46]. Furthermore, available studies are mostly done at the health care level, investigating resistance levels in ESBL-producing *E. coli* and *Klebsiella* spp. isolates [46] or in febrile children younger than 5 years [47]. Few studies exist on ESBL-producing *E. coli* and *Klebsiella* spp. in livestock; however, these have been conducted in urban settings such as Ouagadougou in Burkina Faso [48]. Data on AMR bacteria and their genotype distribution in meat are still scarce especially in rural areas of Ghana and Burkina Faso, for example. In our study, strains will be genotyped to describe phylogenies, mechanisms of resistance, and virulence. Data collected from livestock can also be compared with a human strain collection of an ongoing AMR surveillance system in the 2 districts where the study is implemented in order to describe transmission reservoirs in these parts of Africa.

Anthropological studies will add to the understanding of antibiotic access and utilization patterns, perceptions, and uses of antibiotics among human and animal health care workers, communities, livestock farmers, medicines suppliers, and institutional actors. It will also identify and outline the constraints and enablers of these actors and how these factors influence their behavior. The socioepidemiology data collected among health care workers will generate knowledge on their perception of AMR and the logic of their prescription patterns, while the household survey will quantify community members’ knowledge of antibiotics, AMR, and antibiotic consumption practices. The anthropology and socioepidemiology perspectives will enable a description of the underlying infrastructure and the social, cultural, economic, and political structures that impact the development and spread of AMR. Thereby, this study will add to available literature on health care–seeking behaviors outside of health care facilities, antibiotic use across different levels of health care providers, and access among community members in Burkina Faso [49-51]. Furthermore, by combining the microbiological data on AMR with the perception of institutional actors at different levels, community members, and farmers, we will be able to develop a holistic description of the situation in the 2 study sites. This will represent a necessary step to develop an intervention reflecting the local context and needs.

### Phase 2

The second phase of the project will result in the development of an intervention using the design thinking approach, which is increasingly used in global health to apply social innovation for diverse health issues representing “both a social need and a social problem” [52]. A scoping review indicated that design thinking is used within various initiatives of different organizations and institutions to improve human health [52]. Design thinking is iterative, measurable, and result-driven and places the people being served in the center of the design, innovation, and implementation process [42,43]. Therefore, stakeholders’ actual needs and expectations are met, and acceptable, equitable, effective, and sustainable health solutions can be developed through this active engagement [53-55]. Design thinking consists of stakeholder and cross-disciplinary collaboration and a multistep approach to gain an understanding of the situation and context, brainstorm, test, and revise the best solution in a collaborative process [53]. Therefore, the following steps are repeated over time: define, ideate, prototype, test, and implement [41].

The mixed methods approach and subsequent triangulation of research results will contribute to increasing knowledge and developing a holistic understanding of AMR, which is essential for the development of appropriate interventions at the study sites. Using the One Health perspective, both human and animal health will be examined in this study. The One Health approach will allow us to gain insights into the interconnection of the 2 sectors and to develop a holistic intervention that takes different perspectives and needs into account. Therefore, to follow the One Health paradigm, collaboration across disciplines will be promoted, and actors from different professions and social levels, particularly community members, will be encouraged to bring forward their ideas for solutions. By challenging disciplinary boundaries and through the collaboration of different disciplines within the research team itself and the design teams during the intervention phase, synergy effects and strengths can be brought together to capitalize on innovative potentials. This will be facilitated by a design thinking approach to support innovative potential through the close collaboration of stakeholders and community members, both directly and indirectly affected by AMR. The stakeholder-driven and participatory bottom-up approach to the multifactorial problem of AMR has the potential to result in a common understanding of needs and problems and the subsequent development of a common solution that is tailored to the local situation, context, and needs. Furthermore, it has been shown that moving away from a traditional top-down to a value-driven and participatory bottom-up approach could lead to increased efficacy and acceptability of interventions and improved antibiotic use [26].

In addition, continuous contact with policymakers and the national AMR networks in Burkina Faso and Ghana from the beginning of the research process has the potential to increase the acceptability and scale-up of the resulting intervention that will be developed. By engaging these stakeholders, existing networks, policies, and projects will be incorporated from the first to the last phase of the design thinking approach, which will be built on existing structures and approaches to ensure acceptability, efficacy, and sustainability of the resulting intervention. Moreover, a south-south collaboration will be facilitated within the research project through sharing of competencies and technical know-how within and between the researchers and AMR networks via regular exchanges and through research and dissemination meetings.

### Limitations and Further Considerations

The main limitation of this study is the limited involvement of the environmental sector. The influence of environmental factors on AMR has not been highlighted within the scope of this study. Currently no data have been collected from environmental sources to add to the human and animal data sources. However, we intend to complement this research with such data (collaborations are currently being developed to add this component). In addition, environmental stakeholders from the local and national levels will be included in the qualitative interviews and the development of interventions in the 2 countries.

A diversity of approaches to AMR exists, ranging from research and development of new antibiotics; antibiotic stewardship; improved hygiene; and infection, prevention, and control to behavior change and education of patients and health care professionals. However, social scientists have identified a problematic focus on the causes of AMR linked to the behavior of individuals, both consumers and prescribers, that depoliticizes AMR and isolates it from our modes of life and economies, thereby not acknowledging that AMR is not only a natural phenomenon but also a social fact [56]. Therefore, social scientists urge us to question our way of living in the world, caring for it, and taking care of it [56]. We acknowledge that AMR is a complex and multifaceted issue that needs to be addressed at different levels, taking social and systemic factors into account. For this study, we decided to focus on the behavioral aspects, as these are proven to be an important factor contributing to AMR and lie within the scope of our community-based and participatory approach to develop an intervention. Nonetheless, systemic challenges might become clear within the course of our project and provide important insights for future research projects and policy and decision makers.

Although this study’s multidisciplinary approach offers innovative potential through the close collaboration of different disciplines during continuous (online) meetings and design thinking workshops, it will also present a challenge due to different perspectives, epistemologies, and methodologies. Furthermore, considering current realities and power relations within science, between disciplines and institutions in the so called “Global North” and the “Global South,” we acknowledge that the approach we are promoting through this work, although bottom-up and participatory through actively including community members and other stakeholders, can be challenged from many points of view. Therefore, this study will provide useful insights on how to mitigate such challenges and ensure equitable collaboration.

The generated results will be primarily relevant to the districts of the research project. However, due to direct contact with local, regional, and national stakeholders, the developed intervention can serve as a pilot project for a subsequent scale-up within Burkina Faso and Ghana, as well as other countries. It is expected that these interventions will contribute to improving the way the issue of AMR is addressed among communities, health care providers, livestock farmers, and institutional actors in LMIC. If the interventions in Kossi Province and Asante Akim North District provide positive results, the potential scale-up will be supported by the research team. To facilitate this process, national stakeholders will be informed and engaged right from the project’s inception. The results of this study will provide rigorous scientific evidence on the prevalence of AMR along the dominant food production chains and the perspectives and understanding of AMR among community members and stakeholders from the animal, environmental, and human health sectors in the 2 districts in Burkina Faso and Ghana. Furthermore, insights will be provided regarding the development of an AMR intervention through a participatory bottom-up approach using the design thinking process and the multidisciplinary One Health perspective.

## Appendix

Multimedia Appendix 1 Interview guide in intensive or semi-intensive livestock farms.

Multimedia Appendix 2 Interview guide in villages for human and animal health.

Multimedia Appendix 3 Observation guide and open discussion with households in the villages for human health and animal health.

Multimedia Appendix 4 In-depth interview questionnaire for health care workers in the Ashanti region, Ghana.

Multimedia Appendix 5 IDI Interview Guide for Farmers in Asante Akim North Municipality.

Multimedia Appendix 6 IDI Interview Guide for Community Members in Asante Akim North Municipality.

Multimedia Appendix 7 Interview Guide (Institutional) Actors AMR – Human Health.

## Abbreviations

AMR: antimicrobial resistance
COM-B: capacity, opportunity, motivation, behavior
CSRN: Centre de Recherche en santé de Nouna
ESBL: extended-spectrum beta-lactamase
GLASS: Global Antimicrobial Resistance and Use Surveillance System
KAP: knowledge, attitudes, and practices
LMIC: low- and middle-income countries
WHO: World Health Organization

## Data Availability

The data sets used and analyzed during this study will be available from the corresponding author on reasonable request.

## References

[1] O'Neill J (2016). Tackling Drug-Resistant Infections Globally: Final Report and Recommendations. Review on Antimicrobial Resistance.

[2] Fair RJ, Tor Y (2014). Antibiotics and bacterial resistance in the 21st century. Perspect Medicin Chem.

[3] Prestinaci F, Pezzotti P, Pantosti A (2015). Antimicrobial resistance: a global multifaceted phenomenon. Pathogens and Global Health.

[4] Murray CJ, Ikuta KS, Sharara F, Swetschinski L, Robles Aguilar G, Gray A, Han C, Bisignano C, Rao P, Wool E, Johnson SC, Browne AJ, Chipeta MG, Fell F, Hackett S, Haines-Woodhouse G, Kashef Hamadani BH, Kumaran EAP, McManigal B, Achalapong S, Agarwal R, Akech S, Albertson S, Amuasi J, Andrews J, Aravkin A, Ashley E, Babin F, Bailey F, Baker S, Basnyat B, Bekker A, Bender R, Berkley Ja, Bethou A, Bielicki J, Boonkasidecha S, Bukosia J, Carvalheiro C, Castañeda-Orjuela C, Chansamouth V, Chaurasia S, Chiurchiù S, Chowdhury F, Clotaire Donatien R, Cook Aj, Cooper B, Cressey Tr, Criollo-Mora E, Cunningham M, Darboe S, Day Npj, De Luca M, Dokova K, Dramowski A, Dunachie Sj, Duong Bich T, Eckmanns T, Eibach D, Emami A, Feasey N, Fisher-Pearson N, Forrest K, Garcia C, Garrett D, Gastmeier P, Giref Az, Greer Rc, Gupta V, Haller S, Haselbeck A, Hay Si, Holm M, Hopkins S, Hsia Y, Iregbu Kc, Jacobs J, Jarovsky D, Javanmardi F, Jenney Awj, Khorana M, Khusuwan S, Kissoon N, Kobeissi E, Kostyanev T, Krapp F, Krumkamp R, Kumar A, Kyu Hh, Lim C, Lim K, Limmathurotsakul D, Loftus Mj, Lunn M, Ma J, Manoharan A, Marks F, May J, Mayxay M, Mturi N, Munera-Huertas T, Musicha P, Musila La, Mussi-Pinhata Mm, Naidu Rn, Nakamura T, Nanavati R, Nangia S, Newton P, Ngoun C, Novotney A, Nwakanma D, Obiero Cw, Ochoa Tj, Olivas-Martinez A, Olliaro P, Ooko E, Ortiz-Brizuela E, Ounchanum P, Pak Gd, Paredes Jl, Peleg Ay, Perrone C, Phe T, Phommasone K, Plakkal N, Ponce-de-Leon A, Raad M, Ramdin T, Rattanavong S, Riddell A, Roberts T, Robotham Jv, Roca A, Rosenthal Vd, Rudd Ke, Russell N, Sader Hs, Saengchan W, Schnall J, Scott Jag, Seekaew S, Sharland M, Shivamallappa M, Sifuentes-Osornio J, Simpson Aj, Steenkeste N, Stewardson Aj, Stoeva T, Tasak N, Thaiprakong A, Thwaites G, Tigoi C, Turner C, Turner P, van Doorn Hr, Velaphi S, Vongpradith A, Vongsouvath M, Vu H, Walsh T, Walson Jl, Waner S, Wangrangsimakul T, Wannapinij P, Wozniak T, Young Sharma Temw, Yu Kc, Zheng P, Sartorius B, Lopez Ad, Stergachis A, Moore C, Dolecek C, Naghavi M (2022). Global burden of bacterial antimicrobial resistance in 2019: a systematic analysis. The Lancet.

[5] Brown N (2019). Biotic Politics: Immunitary Imaginaries in Antimicrobial Resistance (AMR). Immunitary Life.

[6] Overton K, Fortané Nicolas, Broom A, Raymond S, Gradmann C, Orubu ESF, Podolsky SH, Rogers Van Katwyk S, Zaman MH, Kirchhelle C (2021). Waves of attention: patterns and themes of international antimicrobial resistance reports, 1945-2020. BMJ Glob Health.

[7] Hinchliffe S, Butcher A, Rahman MM (2018). The AMR problem: demanding economies, biological margins, and co-producing alternative strategies. Palgrave Commun.

[8] Legido-Quigley H, Khan MS, Durrance-Bagale A, Hanefeld J (2018). Something borrowed, something new: a governance and social construction framework to investigate power relations and responses of diverse stakeholders to policies addressing antimicrobial resistance. Antibiotics (Basel).

[9] Collignon P, Beggs JJ, Walsh TR, Gandra S, Laxminarayan R (2018). Anthropological and socioeconomic factors contributing to global antimicrobial resistance: a univariate and multivariable analysis. The Lancet Planetary Health.

[10] Holmes AH, Moore LSP, Sundsfjord A, Steinbakk M, Regmi S, Karkey A, Guerin PJ, Piddock LJV (2016). Understanding the mechanisms and drivers of antimicrobial resistance. Lancet.

[11] Sivaraman GK, Rajan V, Vijayan A, Elangovan R, Prendiville A, Bachmann TT (2021). Antibiotic resistance profiles and molecular characteristics of extended-spectrum beta-lactamase (ESBL)-producing Escherichia coli and Klebsiella pneumoniae isolated from shrimp aquaculture farms in Kerala, India. Front Microbiol.

[12] Sunarno S, Puspandari N, Fitriana F, Nikmah UA, Idrus HH, Panjaitan NSD (2023). Extended spectrum beta lactamase (ESBL)-producing Escherichia coli and Klebsiella pneumoniae in Indonesia and South East Asian countries: GLASS Data 2018. AIMS Microbiol.

[13] Tarrant C, Krockow EM, Nakkawita WMID, Bolscher M, Colman AM, Chattoe-Brown E, Perera N, Mehtar S, Jenkins DR (2020). Moral and contextual dimensions of "inappropriate" antibiotic prescribing in secondary care: a three-country interview study. Front Sociol.

[14] Hedman HD, Vasco KA, Zhang L (2020). A review of antimicrobial resistance in poultry farming within low-resource settings. Animals (Basel).

[15] Lhermie G, Wernli D, Jørgensen PS, Kenkel D, Tauer LW, Gröhn YT (2019). Global resistance to antimicrobials and their sustainable use in agriculture. The Lancet Planetary Health.

[16] Manyi-Loh C, Mamphweli S, Meyer E, Okoh A (2018). Antibiotic use in agriculture and its consequential resistance in environmental sources: potential public health implications. Molecules.

[17] Tadesse BT, Ashley EA, Ongarello S, Havumaki J, Wijegoonewardena M, González Iveth J, Dittrich S (2017). Antimicrobial resistance in Africa: a systematic review. BMC Infect Dis.

[18] (2022). Mapping AMR and AMU Partnership (MAAP). Incomplete Antimicrobial Resistance (AMR) Data in Africa: The Crisis Within The Crisis. African Society for Laboratory Medicine.

[19] Kariuki S, Kering K, Wairimu C, Onsare R, Mbae C (2022). Antimicrobial resistance rates and surveillance in sub-Saharan Africa: where are we now?. IDR.

[20] Escher NA, Muhummed AM, Hattendorf J, Vonaesch P, Zinsstag J (2021). Systematic review and meta-analysis of integrated studies on antimicrobial resistance genes in Africa-A One Health perspective. Trop Med Int Health.

[21] Sariola S, Butcher A, Cañada Jose A, Aïkpé Mariette, Compaore A (2022). Closing the GAP in antimicrobial resistance policy in Benin and Burkina Faso. mSystems.

[22] Tompson AC, Chandler CIR (2021). Addressing antibiotic use: insights from social science around the world. London School of Hygiene & Tropical Medicine.

[23] Vikesland P, Garner E, Gupta S, Kang S, Maile-Moskowitz A, Zhu N (2019). Differential drivers of antimicrobial resistance across the world. Acc Chem Res.

[24] Kakkar M, Sharma A, Vong S (2017). Developing a situation analysis tool to assess containment of antimicrobial resistance in South East Asia. BMJ.

[25] Rogers Van Katwyk S, Hoffman SJ, Mendelson M, Taljaard M, Grimshaw JM (2020). Strengthening the science of addressing antimicrobial resistance: a framework for planning, conducting and disseminating antimicrobial resistance intervention research. Health Res Policy Syst.

[26] Kirchhelle C, Atkinson P, Broom A, Chuengsatiansup K, Ferreira JP, Fortané Nicolas, Frost I, Gradmann C, Hinchliffe S, Hoffman SJ, Lezaun J, Nayiga S, Outterson K, Podolsky SH, Raymond S, Roberts AP, Singer AC, So AD, Sringernyuang L, Tayler E, Rogers Van Katwyk S, Chandler CIR (2020). Setting the standard: multidisciplinary hallmarks for structural, equitable and tracked antibiotic policy. BMJ Glob Health.

[27] Cox J, Vlieghe E, Mendelson M, Wertheim H, Ndegwa L, Villegas M, Gould I, Levy Hara G (2017). Antibiotic stewardship in low- and middle-income countries: the same but different?. Clin Microbiol Infect.

[28] Sié Ali, Louis V, Gbangou A, Müller Olaf, Niamba L, Stieglbauer G, Yé Maurice, Kouyaté Bocar, Sauerborn R, Becher H (2010). The Health and Demographic Surveillance System (HDSS) in Nouna, Burkina Faso, 1993-2007. Glob Health Action.

[29] Wasko Z, Dambach P, Kynast-Wolf G, Stieglbauer G, Zabré Pascal, Bagagnan C, Schoeps A, Souares A, Winkler V (2022). Ethnic diversity and mortality in northwest Burkina Faso: an analysis of the Nouna health and demographic surveillance system from 2000 to 2012. PLOS Glob Public Health.

[30] (2022). Burkina Faso national action plan on antimicrobial resistance: review of progress in the human health sector. World Health Organization.

[31] Asante Akim North Municipal Assembly.

[32] (2019). Republic of Ghana Composite Budget for 2019-2022, Programme Based Budget Estimates for 2019, Asante Akim North District Assembly. Ministry of Finance and Economic Planning (Ghana).

[33] (2022). Global antimicrobial resistance and use surveillance system (‎GLASS)‎ report: 2022. World Health Organization.

[34] (2021). Global Antimicrobial Resistance and Use Surveillance System (GLASS) Report: 2021. World Health Organization.

[35] Koduah A, Gyansa-Lutterodt M, Hedidor GK, Sekyi-Brown R, Asiedu-Danso M, Asare BA, Ackon AA, Annan EA (2021). Antimicrobial resistance national level dialogue and action in Ghana: setting and sustaining the agenda and outcomes. One Health Outlook.

[36] Hein W, Aglanu LM, Mensah-Sekyere M, Harant A, Brinkel J, Lamshöft Maike, Lorenz E, Eibach D, Amuasi J (2022). Fighting antimicrobial resistance: development and implementation of the Ghanaian National Action Plan (2017-2021). Antibiotics (Basel).

[37] (2017). Ghana: National action plan for antimicrobial use and resistance: 2017-2021. World Health Organization.

[38] Opintan JA (2018). Leveraging donor support to develop a national antimicrobial resistance policy and action plan: Ghana's success story. Afr J Lab Med.

[39] Michie S, van Stralen Maartje M, West R (2011). The behaviour change wheel: a new method for characterising and designing behaviour change interventions. Implement Sci.

[40] Gibbons S (2016). Design Thinking 101. NN/g Nielsen Norman Group.

[41] Kreitzer MJ, Carter K, Coffey DS, Goldblatt E, Grus CL, Keskinocak P, Klatt M, Mashima T, Talib Z, Valachovic RW (2019). Utilizing a systems and design thinking approach for improving well-being within health professions’ education and health care. NAM Perspectives.

[42] Brown T, Wyatt J (2015). Design thinking for social innovation. Annual Review of Policy Design.

[43] Catalani C, Green E, Owiti P, Keny A, Diero L, Yeung A, Israelski D, Biondich P (2014). A clinical decision support system for integrating tuberculosis and HIV care in Kenya: a human-centered design approach. PLoS One.

[44] Dodd S, Widnall E, Russell AE, Curtin EL, Simmonds R, Limmer M, Kidger J (2022). School-based peer education interventions to improve health: a global systematic review of effectiveness. BMC Public Health.

[45] Butcher A, Rahman MM (2022). Innovation at the margins: the challenges for Bangladesh's aquaculture hatchery sector. The International Journal of Sociology of Agriculture and Food.

[46] Garba Z, Kaboré Bérenger, Bonkoungou IJO, Natama MH, Rouamba T, Haukka K, Kirveskari JP, Tinto H, Sangaré Lassana, Barro N, Kantele A (2023). Phenotypic detection of carbapenemase and AmpC-β-lactamase production among extended spectrum β-lactamase (ESBL)-producing Escherichia coli and Klebsiella spp. isolated from clinical specimens. Antibiotics (Basel).

[47] Bonko MDA, Tahita MC, Kiemde F, Lompo P, Yougbaré Sibidou, Some AM, Tinto H, Mens PF, Menting S, Schallig HDFH (2021). Antibiotic susceptibility profile of bacterial isolates from febrile children under 5 years of age in Nanoro, Burkina Faso. Trop Med Int Health.

[48] Soma D, Bonkoungou IJO, Garba Z, Diarra FBJ, Somda NS, Nikiema MEM, Bako E, Sore S, Sawadogo N, Barro N, Haukka K (2024). Extended-spectrum beta-lactamase-producing and multidrug-resistant Escherichia coli and Klebsiella spp. from the human–animal–environment interface on cattle farms in Burkina Faso. Microbiology Research.

[49] Compaoré Adélaïde, Rouamba T, Kaboré Bérenger, Jacobs J, Peeters Grietens K, Sariola S (2024). Exploring antibiotic use in the community: a household-based survey using the drug bag method in rural Burkina Faso. Antibiotics (Basel).

[50] Valia Daniel, Ingelbeen Brecht, Nassa Guétawendé Job Wilfried, Kaboré Bérenger, Kiemdé François, Rouamba Toussaint, Compaoré Adélaïde, Kouanda Juste Stéphane, Robert Annie, Rodriguez-Villalobos Hector, Van Der Sande Marianne A B, Tinto Halidou (2024). Antibiotic use by clinical presentation across all healthcare providers in rural Burkina Faso: a healthcare visit exit survey. J Antimicrob Chemother.

[51] Valia D, Kouanda JS, Ingelbeen B, Derra K, Kaboré Bérenger, Kiemdé François, Rouamba T, Rouamba E, Hien FS, Campbell L, Meudec M, Robert A, Tinto H, van der Sande MAB, Villalobos HR (2023). Healthcare seeking outside healthcare facilities and antibiotic dispensing patterns in rural Burkina Faso: a mixed methods study. Trop Med Int Health.

[52] Bazzano AN, Martin J, Hicks E, Faughnan M, Murphy L (2017). Human-centred design in global health: a scoping review of applications and contexts. PLoS One.

[53] Hendricks S, Conrad N, Douglas TS, Mutsvangwa T (2018). A modified stakeholder participation assessment framework for design thinking in health innovation. Healthc (Amst).

[54] Manzini E, Coad R (2015). Design, When Everybody Designs: An Introduction to Design for Social Innovation.

[55] Altman M, Huang TT, Breland JY (2018). Design thinking in health care. Prev Chronic Dis.

[56] Varobieff L, Harpet C (2022). Chapitre 10 - L’antibiorésistance, une invitation à repenser la relation. L'antibiorésistance: un fait social total.

