# Supplementary material for: Exploring the Current Situation and Developing Strategies for Behavior Change to Improve Antibiotic Use in West Africa: Protocol for a Multidisciplinary Interventional Research Project
Source: JMIR Res Protoc. 2025 Jul 25;14:e66424. doi: 10.2196/66424 (PMC12334893; doi:10.2196/66424)
Supplement: Multimedia Appendix 1 [file resprot_v14i1e66424_app1.docx]

Phase 1.2. – anthropology

**Interview guide in intensive or semi-intensive livestock farms**

*This interview guide will be consolidated after the first observations are made. We will start with observation and free interviews before conducting semi-structured interviews.*

*These interviews will be conducted by the junior researcher, the post-doctoral fellow and Carine Baxerres, during the second half of the ethnographies of the farms. They will be conducted with the staff of the farms.*

*Written consent will be obtained beforehand (see information and consent forms).*

Starting question: To begin with, I would like you to tell me about your professional career up to now and this farm in which you work. Did you have an education? What other jobs have you had before? How did you get started here?

**1) Professional background**

- Schooling, secondary studies, continuing education

- the different activities and jobs occupied

**2) Work on the farm**

Can you tell me about the farm where you work? What is its history? How did it evolve? Can you also tell me how you manage the farm? How do you do it? What are the tasks you have to perform?

- History of the farm and its evolution over time. Is it linked to a larger structure? Where does the capital come from? Are there shareholders? Where do the animals come from initially?

- Tasks performed by the respondent.

- Where does the feed for the animals come from? What other inputs are used on the farm?

- Difficulties encountered.

- Relationships among farm personnel.

- Strategies for developing the productivity of the farm. If products are used, ask which ones, why, and where they were purchased. Insist particularly if antibiotics are used (molecules, commercial names, prices).

- Who are the customers and how are they sold?

- How is the waste from the farm managed?

**3) Animal health management**

- What animal health problems are encountered? How do they deal with them?

- Do they use medication or other products to prevent and treat animal diseases? Which ones, bought where, at what price? How do they know about these products? How do they give them to the animals (dosages, method of administration)? Do people come by to promote and sell these products to the farm?

- Do they use animal health professionals? In what cases? To whom? Do they travel to them or do they come? Where are they based? How much does it cost? What do they advise them to use in terms of medicines or other products? Where do they buy these products? How much do they charge?

- What do they do with the medicines or other products they use? Where are they stored? Who manages this place? What happens to products that are out of date or that they want to get rid of?

**4) Uses and perceptions of antibiotics**

- Go back to the different antibiotics mentioned and try to find out for what health issues these products are given to animals? or ask if they use antibiotics for animals? If so, which ones and how are they given (dosage)?

- Where are these products bought, how much are they bought for, why are they bought there?

- What is the difference between these different products?

- What does he think about antibiotics in general for animals (effectiveness, old and new products, side effects, for what health issues)? How does he/she know all this about antibiotics for animals?

- Do they use herbal medicine (standardized or not) as an alternative to antibiotics for animals? Who advised them? Where do they buy it?

- *Did the issues of antibiotic resistance come up in the previous discussion and if not* is he aware of this issue *(to be phrased in a way that the person understands without using this term)*, if so where did he learn about it, what does he think about it?

**5) Discussion of the farm's pharmacy**

- *Is there a pharmacy on the farm and if so,* is it possible to see it and discuss together about the products it contains? (Take one by one the different products and tell each other why it was bought, where, how much, why it can be used too, etc.)

*If possible, take a picture of the pharmacy and where it is stored. List (on a sheet/book) the medicines in the pharmacy, note the molecules, the marketing names, the countries of manufacture, the producers, the wholesalers, describe the packaging; and extend the discussion from the interview.*

*Observe how the medicines are stored, what the pharmacy looks like, where it is stored, how the medicines are kept, what aspects they have (dirty, damaged...), etc.*

Characteristics of the respondent *(to be filled in at the end of the interview, once the recorder is turned off, for information that will not be revealed during the interview)*

Level of education :

Age:

Renter/Lives in a relative's house/Owner:

Average monthly income: (**From Observation, try to put the person in a category, ‘rich’, ‘middle class’, ‘poor’)**

Mother tongue/sociolinguistic group/geographical origin (where was he/she born):

Religion, if Christian specify church:

Owns a vehicle? if yes, which one(s):
