# Supplementary material for: Exploring the Current Situation and Developing Strategies for Behavior Change to Improve Antibiotic Use in West Africa: Protocol for a Multidisciplinary Interventional Research Project
Source: JMIR Res Protoc. 2025 Jul 25;14:e66424. doi: 10.2196/66424 (PMC12334893; doi:10.2196/66424)
Supplement: Multimedia Appendix 2 [file resprot_v14i1e66424_app2.docx]

Phase 1.2. – anthropology

**Interview guide in villages for human and animal health**

*This interview guide will be consolidated after the first observations are made. We will start with observation and free interviews before conducting semi-structured interviews.*

*These interviews will be conducted by the junior researcher during the second half of his/her immersion in the villages. They will be conducted with 30 people: men and women of varying ages (young adults, middle-aged adults, elderly people) and different socio-economic status ("rich", "middle class", "poor"), some of whom will have dependent children (aged 0 to 15). Twenty of these individuals will be engaged in food crop production, while the other 10 will not (if this is the case for some in the villages studied).*

*Oral consent will be obtained prior to the interview, with the signature of a third party (see information and consent forms).*

**Interview Part 1: Human Health**

Starting question: To begin, I would like you to tell me how you deal with health issues in your household, your own, your children's, your spouse's (if any). What health problems do you have most often? What do you do initially when it starts? What do you do afterwards if it continues? What products do you use? What professionals might you see? What else might you be doing to stay healthy or not get sick? I'm going to take the first name of each member of the household (people eating from the same pot) and you're going to tell me about the last time each of you had to do something related to health (take a product, go to a doctor, go to a traditional therapist, maybe pray too, etc.), maybe for an illness or more generally to maintain good health, maybe after having gone to a doctor or on your own initiative or under the advice of someone you know So let's start with...

**1) Daily health management**

- What are their preventive, curative and health maintenance self-medication practices? With what types of products (pharmaceutical drugs, standardized and non-standardized herbal products, others), purchased where, at what price, prepared by whom, found where, advised by whom, dosages of use?

- What are the health consultation practices? What types of professionals (biomedical (Pulic or private), "traditional", religious, other), chosen how, at what price, what prescriptions, bought where, at what price?

- To be told about the therapeutic itineraries and the meaning put behind these itineraries.

- Does he/she ever throw away medicines? In what cases? Where and how?

**2) Uses and perceptions of antibiotics**

- Go back to the different antibiotics mentioned and try to find out for what health issues this product is used, how it is taken (dosage), can all members of the household use it?

- Where are these products purchased, how much are they purchased for, why are they purchased there?

- What is the difference between these different products?

- What does he/she think about antibiotics in general (effectiveness, old and new products, side effects, for what health issues)? How does he/she know all this about antibiotics?

- Does he/she use herbal medicine (standardized or not) as an alternative to antibiotics? Who advised him/her? Where does he/she buy it?

- *Did the issues of antibiotic resistance come up in the previous discussion and if not,* is he/she aware of this issue *(to be formulated so that the person understands without using this term)*, if so where did he/she learn about it, what does he/she think about it, ?

**3) Discussion about the home pharmacy (This should be done after the interview). Infomation derived should be written in the word file after the interview.**

- *Does he/she have a home pharmacy and if so,* is it possible to see it and discuss together about the products it contains? (take one by one the different products and have them tell you why it was bought, where, how much, why can it be used too, etc.).

*If possible, take a picture of the home medicine cabinet and where it is stored. List (on a sheet/book) the medicines in the home pharmacy, note the molecules, marketing names, countries of manufacture, producers, wholesalers, describe the packaging; and extend the interview discussions by asking about the usefulness of certain medicines and/or why they were used.*

*Observe how medicines are stored, what the home medicine cabinet looks like, where it is stored, whether there are several in the family, how medicines are kept, what they look like (dirty, damaged...), etc.*

**Part 2 of the interview: Animal health (for those who practice food production)**

Starting question: To continue, I would like you to tell me about the way you take care of the animals you raise, especially their health issues? What animals do you raise? How do you handle them in general? What about their health? Do you give them products regularly? Do you ever consult with animal health professionals?

**1) Animal management and health**

- What animals does he/she raise and for what purposes (household consumption, sale, savings, etc.)?

- How does he/she take care of them in general? Can he/she describe a typical day of management of the different animals?

- Does he/she use medication or other products to take care of his/her animals? Which ones (molecules and composition)? For what purposes (production, health, curative, preventive, etc.)? Where are they purchased, how much, from whom, why there?

- How did he/she learn to use these products? Did he/she attend any training? Has he/she received advice and from whom?

- Does he/she receive visits from animal professionals? If yes, which ones and why?

- Does he/she ever go to see animal professionals? If yes, which ones, for what health issues and how? Do they prescribe products? If yes, which ones and where does he/she buy them?

- How does he/she manage the waste from his/her livestock (feces, leftover feed, leftover products)? What do they do with it?

**2) Uses and perceptions of antibiotics**

- Go back to the different antibiotics mentioned and try to find out for what health issues these products are given to the animals? or ask if he/she uses antibiotics for his/her animals? If so, which ones and how are they given (dosage)?

- Where are these products bought, how much are they bought for, why are they bought there?

- What is the difference between these different products?

- What does he/she think about antibiotics in general for animals (effectiveness, old and new products, side effects, for what health issues)? How does he/she know all this about antibiotics for animals?

- Does he/she use herbal medicine (standardized or not) as an alternative to antibiotics for his/her animals? Who advised him/her? Where does he/she buy it?

- What does he/she do with the antibiotics bought for the animals that are outdated or that he/she no longer wants? how does he/she dispose of them?

- *Did the issues of antibiotic resistance come up in the previous discussion and if not,* is he/she aware of this issue *(to be phrased in a way that the person understands without using this term)*, if so where did he/she learn about it, what does he/she think about it?

**3) Discussion about the farm animal pharmacy (This should be done after the interview)**

- *Does he/she have a pharmacy for animals and if so,* is it possible to see it and discuss together about the products it contains? (take one by one the different products and have the person tell why it was bought, where, at what price, why can it be used too, etc.)

*If possible, take a picture of the pharmacy and where it is stored. List (on a sheet/book) the medicines in the pharmacy, note the molecules, the marketing names, the countries of manufacture, the producers, the wholesalers, describe the packaging; and extend the discussion from the interview.*

*Observe how the medicines are stored, what the home pharmacy looks like, where it is stored, whether there are several in the family, how the medicines are kept, what they look like (dirty, damaged...), etc.*

Characteristics of the respondent *(to be filled in at the end of the interview, once the recorder is turned off, for information that will not be revealed during the interview)*

Level of education:

Profession/activities:

Age:

Renter/Lives in a relative's house/Owner:

Average monthly income: (**From Observation, try to put the person in a category, ‘rich’, ‘middle class’, ‘poor’)**

Average monthly household income:

Mother tongue/sociolinguistic group/Geographic origin (where was he/she born):

Religion, if Christian specify church:

Owns a vehicle?, if yes, which one(s):

*Note the socio-economic level of the household after the interview is conducted and the home is observed ("affluent," "intermediate," "impoverished")*
