# Supplementary material for: Exploring the Current Situation and Developing Strategies for Behavior Change to Improve Antibiotic Use in West Africa: Protocol for a Multidisciplinary Interventional Research Project
Source: JMIR Res Protoc. 2025 Jul 25;14:e66424. doi: 10.2196/66424 (PMC12334893; doi:10.2196/66424)
Supplement: Multimedia Appendix 3 [file resprot_v14i1e66424_app3.docx]

Phase 1.2. – anthropology

**Observation guide and open discussion with households in the villages for human health and animal health**

*These observations will be conducted by the junior researcher in two villages in both countries. The villages will be part of the areas studied in WP1 of the project. They will be distant from each other and will be chosen according to the animals involved in food production (poultry, cattle, sheep) and the types of health centers present locally (public and private, at different levels of the health pyramid), in order to observe contrasting situations. In Burkina Faso, we will work in two villages in the department of Nouna and in Ghana, in two villages in the Ashanti region near the town of Agogo.*

*The junior researcher will be housed in each of these villages for one month, if possible in a family that practices livestock production.*

*As many families/households as possible will be observed on these topics. During the observations, open discussions will be held with the people present on the topics of interest in the study.*

*Oral consent will be obtained beforehand, with a third party signature (see information and consent forms).*

**1) Human health**

- What are the different drug distributors (public, private, informal) for human health in the villages studied or nearby?

- What are the different health centers in or near the villages studied?

- What are the hygiene practices in the family? Are they facilitated or not?

- What are the self-medication practices (either from the person alone, or from his family environment, friendship environment) in the households and what meaning do people give to these practices for children, for adults, for the elderly?

- -Who are the people with whom health issues are discussed? the decisions taken?

- Are antibiotics involved in these self-medication practices, which ones (molecules, marketing name, country of manufacture, producers, wholesalers, packaging) and for what health issues? Where are they purchased and at what price? Who paid and how was the money found? How are they taken and in what dosage?

- Do households have home pharmacies and if so, what are their contents? If so, a description should be provided.

- What are the consultation practices of the households, with which health centers/health professionals (biomedical, such as "traditional," religious, spiritual, informal) and for which health issues for children, for adults, for the elderly?

- Are antibiotics involved in these prescriptions, which ones (molecules, marketing name, country of manufacture, producers, wholesalers, packaging) and for which health issues? Where are they purchased after the prescription and at what price? Who paid and how was the money found? How are they taken and according to what dosage?

- Are alternative phytomedicine products to antibiotics used in self-medication or following a prescription? If so, which ones (molecules, marketing name, country of manufacture, producers, wholesalers, packaging) and for what health issues? Where are they purchased and at what price? How are they taken and in what dosage?

- Are there differences in care depending on the members of the family (children, adults, working men, working women, pregnant women, elderly etc.)?

- What are people's perceptions of the different antibiotics they encounter?

- How are expired medications or medications that people want to get rid of managed? Where are they disposed of and how?

- Where do people relieve themselves? Is it in nature or in a bathroom?

- Do people know about antibiotic resistance as it relates to human health? If so, what do they know about it and how did they learn about it?

**2) Animal health**

- What are the different drug distributors (public, private, informal) for animal health in the villages studied or nearby?

- Do all families/households in the villages raise food crops, or do only some people do so?

- What types of animals are raised in this way?

- What are the hygiene practices in the family around animals? Are they facilitated or not?

- Are medicines given to these animals for productivity or in relation to animal health issues? If so, which ones? Are there antibiotics among them and if so which ones (molecules, marketing name, country of manufacture, producers, wholesalers, packaging) and for what health issues? Where are they purchased and at what price? How are they given and in what dosage?

- Do people have pharmacies for the health of their animals and if so, what is their composition? If so, a description should be given.

- Are alternative phytomedicine products to antibiotics used for the animals raised? If yes, which ones (molecules, marketing name, country of manufacture, producers, wholesalers, packaging) and for what health issues? Where are they purchased and at what price? How are they given and in what dosages?

- Do people use animal health professionals and if so which ones? Do these professionals travel or do people go to them? If they travel, how are the consultations conducted? Do these professionals recommend antibiotics? If yes, which ones, where do they buy them, at what price? How are they given to animals and in what dosage?

- What are people's perceptions of the different antibiotics encountered in animal health?

- How are outdated medications or medications that people want to get rid of managed? Where are they disposed of and how?

- Do people have any knowledge about antibiotic resistance in relation to animal health? If so, what do they know and how did they learn about it?

**3) The overall understanding of the village**

- Roughly in the middle of the ethnography (after 5 to 6 weeks), try to draw a diagram of the village precisely and the issues in the village (related to health or not, indicate the places where for the people and animals to wash, waste management, places of power, places of worship, tontine / microcredit groups, places of distribution of products, market, circulation of water, circulation of sellers ...) with people with which the researcher interacted the most with. And take a picture of this diagram.
