# Supplementary material for: Exploring the Current Situation and Developing Strategies for Behavior Change to Improve Antibiotic Use in West Africa: Protocol for a Multidisciplinary Interventional Research Project
Source: JMIR Res Protoc. 2025 Jul 25;14:e66424. doi: 10.2196/66424 (PMC12334893; doi:10.2196/66424)
Supplement: Multimedia Appendix 4 [file resprot_v14i1e66424_app4.docx]

**Phase 1.3. – socio-epidemiology qualitative**

**In-depth Interview questionnaire for health care workers in the Ashanti Region, Ghana**

| **Respondent ID** |  |
| --- | --- |
| Date of Interview |  |
| Age |  |
| Gender |  |
| Professional qualification |  |
| Years of work experience |  |
| Years of work experience in this health facility |  |
| Type of health facility |  |
| Location |  |

**Interview guideline**

**Narrative:**

***In-depth interview with health care workers in the Ashanti Region of Ghana on the perception of antibiotic resistance and their prescription habits.***

This is a qualitative study designed to look into the understanding and perception of antibiotic resistance among the health care workers in the Ashanti Region in Ghana. The aim is to explore the current knowledge on the topic of antibiotic resistance and how it translates into prescription habits. We aim to identify barriers to the responsible use of antibiotics and the thoughts of health care workers on how to overcome them.

Interviews will be conducted in at least 3 different health facilities in the Ashanti Region with medical doctors, physician assistants and nurses trained in prescription of antibiotics with at least 1 year work experience. The number of interviews will depend on the point when saturation is reached. The results will form part of the ground research to develop interventions adapted to the context to reduce antibiotic resistance.

**Overall Objectives:**

- To document the factors that influence health care workers during the prescription of antibiotics
- To understand the perception of antibiotic resistance of health care workers in the Ashanti Region in Ghana
- To provide ideas on possible solutions to improve the use of antibiotics in the Ashanti Region in Ghana from the health worker perspective

**Specific Objectives:**

- To identify the considerations of health care workers when prescribing antibiotics
- To provide individual perspectives on antibiotic resistance, its contributing factors and the impact on health and well-being
- To relate knowledge about correct antibiotic prescription to actual practice
- To explore the difficulties in the daily work that hinder health care workers to apply strategies to avoid antibiotic resistance
- To determine possible interventions to support the correct use of antibiotics

1. **Greetings and Introduction**

Welcome and thank you for participating in this research.

My name is X, and I come from__. I am part of a team conducting a joint research project from the Heidelberg University in Germany together with Kumasi Centre for Collaborative Research in Tropical Medicine (KCCR) in Kumasi to learn more about how health care workers such as yourself perceive antibiotic resistance and how that influences you in your daily work when you are prescribing antibiotic drugs. The interview will take between 45 and 60 min.

According to the Ethics committees in Germany and Ghana we need your consent to participate in this interview. I think you already signed the informed consent form, but let me ask you again at this moment, if you agree to be part of this study. I want to emphasise again, that we will protect your privacy, no personal or sensitive information will be kept. We will need to record our conversation for facilitating our note taking and to make sure that we follow the conversation correctly. These recordings will be deleted after transcription and your name will never be kept with the recording. Do you agree to participate and for it to be recorded? You can decide to stop your participation at any time without any negative consequence for you. You can also ask to stop the record for some comments you would not have to have recorded. Don’t hesitate to ask for clarification at any point during or after the interview.

At the beginning we would like to ask you to tell us a little bit about yourself and your working experience.

1. **Introduction questions**

| **NOTES FOR THE INTERVIEWER**   - *Fill in the information at the top of the document* |
| --- |

- What is your working experience (in your current position)?
- Have you worked somewhere else before? What was your position then?
- What are the main tasks during your daily work?

1. **Prescription habits for antibiotic drugs**

| **NOTES FOR THE INTERVIEWER**  *Key ideas to explore:*   - *What steps do health care workers follow when they are prescribing antibiotics? Is antibiotic resistance considered in the process?* - *Is the theoretical knowledge about correct use of antibiotics and risks for the development of antibiotic resistance translated into practice? (Context with the following questions)* - *What influences their prescriptions?* - *Do they follow the national policies on which antibiotics they are allowed to prescribe?* |
| --- |

- Can you walk us through the steps of your thought process when deciding on antibiotic treatment and prescribing an antibiotic drug? What are your considerations?
  - How do you decide if somebody needs an antibiotic?
  - How do you decide on the duration and dosage of the drug prescribed?
  - When and how do you decide to stop or change an antibiotic?
  - Which tool do you have available to help you in the decision? – guidelines/electronic resources… are they adapted for different age groups?
  - What role do guideline or algorithms play for antibiotic prescriptions in your practice?
  - Can you think of reasons, why you sometimes do not follow the recommendations?
  - If you are not sure about the antibiotic treatment of a patient, where would you look for help or information? (Possible prompts: guidelines, colleagues, committees)
  - What do you tell patients about the prescription?
- Can you think of situations in which you decided on antibiotic treatment even though you thought it is probably not necessary? If yes, what were the reasons for prescribing antibiotics anyway?
- Can you tell us about situation where people, friends or family approached you outside of your working place and asked you for a prescription?
- Which antibiotics do you use most? What are the reasons for that?
- Can you tell us if there have been situations when you have prescribed antibiotics that are not available in your health facility?
- How do the patients influence the prescription?
  - Can you remember situations when patients were actively demanding antibiotic treatment?
  - Can you remember situations when patients were refusing antibiotic treatment? – What reasons did they have for refusing?
  - What questions do patients ask after you give them the prescription and maybe some explanations?

1. **Association with the “term” Antibiotic Resistance**

| **NOTES FOR THE INTERVIEWER**  It is likely that the study site has been part of a study on AMR before. Avoid the abbreviation AMR. It could be strongly associated with the research done in the laboratory and prevent participants from thinking outside of “bacterial culture and resistance testing”.  *Key ideas to explore:*   - *What do health care workers associate with Antibiotic resistance?* - *Have they heard of the concept before? Where did they learn about it? (school/university/work/other colleagues)* - *How important do they think the topic is?* - *Which causes for Antibiotic Resistance are health care workers are aware of?* |
| --- |

- How would you explain antibiotic resistance?
  - When and how did you first hear about antibiotic resistance?
- What are some of the reason for the appearance of antibiotic resistance?
  - Can you think of any practices that could lead to antibiotic resistance?
  - Apart from the people/groups you mentioned, who might also influence the appearance of antibiotic resistance?
- How important do you think the topic is? What are the dangers/problems with antibiotic resistance?
- How has the situation of Antibiotic Resistance changed since you started your professional career (On a practical level? On an organisational/policy level?)
- How did your personal perception of Antibiotic Resistance change since you started working?
  - Importance of the topic, Responsibility, etc…

1. **Perception of Antibiotic Resistance in daily work**

| **NOTES FOR THE INTERVIEWER**  *Key ideas to explore:*   - *Are health care workers alert to Antibiotic Resistance during their daily work?* - *How do health care workers perceive their responsibility in preventing Antibiotic Resistance?* - *Have they been confronted with problems due to Antibiotic Resistance?* - *Is it a topic that is discussed with colleagues and patients?* - *Do they recognize the importance of infection control?* |
| --- |

- When do you suspect that a patient might suffer from an infection with a resistant bacterium?
- What are your options if you suspect an infection with a resistant pathogen?
- Is there anything in your work that can reduce/increase Antibiotic Resistance? How do you see the importance of infection control inside your health facility?
  - Through which ways can infections spread in a health facility?
  - Can you see links to hygiene or other common practices?
  - What are the measures you take to avoid infections in your workplace?
  - What is the connection between hospital acquired infections and antibiotic resistance?
- Have you ever come across a patient where the standard antibiotic treatment did not work? Can you tell me about it?

1. **Barriers to and challenges for good “Antibiotic Resistance” practices**

| **NOTES FOR THE INTERVIEWER**  Provide the introduction to this topic (see below) to the participant before asking the questions in order to orientate him/her towards the information we want to explore.  *Key ideas to explore:*   - *What is preventing health care workers to translate theoretical knowledge into good “Antibiotic Resistance” practice? Lack of knowledge? Lack of resources/information sources? Disinterest? Practical issues?* |
| --- |

**Introduction to this topic for participant:**

You have told me about your views on antibiotic resistance. We have talked as well about how antibiotic resistance influences your work and the possible causes for resistance. Now I would like to focus on one special cause. **Overuse or inappropriate use of antibiotic drugs** is one of the driving forces for antibiotic resistance. At the very beginning of our talk, you explained the process of prescribing antibiotics when you are working. Now I would kindly ask you to link this knowledge with your practice when answering the last few questions.

**Questions:**

- Earlier you told me what steps you consider when you are prescribing antibiotics and what you can do to reduce antibiotic resistance. Which of these steps are sometimes difficult to follow in your daily work and why?
- Which are some of challenges you face that are related to the patients?
- Could you give us some examples where self-treatment of patients had caused you difficulties?
- Which difficulties do you have in accessing tools/information on antibiotic resistance?
- What are some of the barriers you face that are related to the setting you work in?

1. **Possible interventions**

| **NOTES FOR THE INTERVIEWER**  *Key ideas to explore:*   - *Have health care workers thought about or discussed possible solutions to eliminate barriers of good antibiotic resistance practices?* |
| --- |

- What type of support would you need to help you with the difficult situations with antibiotic prescription or antimicrobial resistance?
  - Can you think of something that would be useful for the difficulties you described?

Can you name a few tools or equipment that you would wish to have? What is your opinion on technology like an app, for example?

- - What are the areas where you would want to receive training? How would you like that training to look like?
  - Who would you wish to get more support from?

1. **End/conclusion of interview**

Thank you very much for sharing your time with us and for answering all of our questions. I would like to give you the opportunity now to **add anything on the topic that you think is important and that I have not specifically asked you**?

In case we have further questions or need some clarifications on the answers you have given, **would you agree that we contact you again**?

Again, thank you so much for your cooperation and your openness. Have a nice day and all the best for you.
