# Supplementary material for: Exploring the Current Situation and Developing Strategies for Behavior Change to Improve Antibiotic Use in West Africa: Protocol for a Multidisciplinary Interventional Research Project
Source: JMIR Res Protoc. 2025 Jul 25;14:e66424. doi: 10.2196/66424 (PMC12334893; doi:10.2196/66424)
Supplement: Multimedia Appendix 5 [file resprot_v14i1e66424_app5.docx]

**Phase 2. – interventional research component**

IDI Interview Guide for Farmers in Asante Akim North Municipality

| **Respondent ID** | ID1 (Community Animal Health) |
| --- | --- |
| Date of interview |  |
| Age |  |
| Gender |  |
| Educational background |  |
| Professional background |  |
| Number animals (specify) |  |
| Location (Konongo) |  |

**Narrative:**

*In-depth interview with community members in the Ashanti Region of Ghana on the understanding and perception of antibiotics and AMR.*

This is a qualitative study designed to look into the understanding and perception of antibiotics and AMR among community members in the Ashanti Region of Ghana. The aim is to explore current knowledge on the topic of antibiotics and AMR, whether AMR is perceived as a problem and if so, what possible solutions against AMR could look like. The aim is to gain insights into perspectives of different community members.

Interviews will be conducted with patients/customers exiting the wholesalers in Konongo and the informal veterinary market in Ananekrom. The number of interviews will be determined by the reaching of saturation. The results will form part of the ground research to develop an AMR intervention adapted to the local context and needs.

**Objectives:**

- To gain insights into farmers’ understanding and perception of antibiotics and AMR.
- To identify possible needs and challenges related to antibiotics and AMR.
- To identify possible solutions/interventions on AMR.

1. **Introduction**

- Introduction of interviewer.
- Explanation of project and interview procedure (participation voluntarily, not paid).
- Signing of informed consent form.

1. **Introduction questions**

- You have just excited the …. Did you get antibiotics?
- If not, do you still remember the last time you used antibiotics for your animals?

1. **. Antibiotics**

- Do you know what antibiotics are and how would you describe them?

*[If the interviewee does not know what antibiotics are show them examples]*

- According to you understanding, what is the purpose of antibiotics?
  - Do you use them for preventive treatment or to cure sick animals?
  - How do you know when your animals need antibiotics?
  - Do you ask anyone for advice in such a case? If so, who?
  - How do they [veterinarians, veterinary technical assistants, others] know if your animals need antibiotics?
  - Can you know from the symptoms if your animals need antibiotics or not? Can the vets, vet technical assistants, or fellow farmers know? How do they know?
- Could you give me any examples of antibiotics you know or already used for your animals?
  - How do you identify whether a drug is an antibiotic? (name, packaging, colour)
- Do you still remember where you bought antibiotics when you last needed them for your animals?
  - Could you please describe the process of getting antibiotics?
  - Is it easy to get antibiotics? Where can you get them?
  - Could you describe any facilitators or barriers you might have experienced when last getting antibiotics?
- What is your experience with antibiotics? (positive/ negative, side effects, efficiency, instructions given, economic implications of adhering to withdrawal period)

1. **AMR**

- Have you heard about AMR already?

*[If the interviewee does not know AMR explain it using Damien’s cartoon]*

- - If yes, where and what?
  - How would you describe AMR?
- According to your opinion, what is causing and driving AMR?
  - Can you think of any practices that can lead to AMR?
    (human medicine, animal breeding, environment)
- How important do you think the topic of AMR is?
  - What are the dangers or problems related to AMR?
  - Does AMR have any consequences to your animals? If so, which?
  - Does AMR have any consequences for you personally or your community? If so, which?
- According to your perception, is AMR discussed in Ghana? If yes, since when and how?
  - Do you think that AMR is national/regional/international problem? Why?

1. **AMR action**

- Have you heard or seen any campaign, pictures, radio messages, or other information about AMR? If so, what was the main message?
- What are do you think could be helpful to improve the situation regarding AMR?
  - Who should be involved in your suggested solutions?
  - Who should be targeted by such an intervention? (stakeholders, sectors)
- What role could you play in possible future action related to AMR?
- What role could your community play in possible future action related to AMR?

1. **End/conclusion of interview**

- Thank for time and valuable answers. Outlook.
