# Supplementary material for: Exploring the Current Situation and Developing Strategies for Behavior Change to Improve Antibiotic Use in West Africa: Protocol for a Multidisciplinary Interventional Research Project
Source: JMIR Res Protoc. 2025 Jul 25;14:e66424. doi: 10.2196/66424 (PMC12334893; doi:10.2196/66424)
Supplement: Multimedia Appendix 6 [file resprot_v14i1e66424_app6.docx]

**Phase 2. – interventional research component**

IDI Interview Guide for Community Members in Asante Akim North Municipality

| **Respondent ID** | ID1 (Community Human Health) |
| --- | --- |
| Date of interview |  |
| Age |  |
| Gender |  |
| Educational background |  |
| Professional background |  |
| Number of children |  |
| Location (Agogo, Ananekrom) |  |

**Narrative:**

*In-depth interview with community members in the Ashanti Region of Ghana on the understanding and perception of antibiotics and AMR.*

This is a qualitative study designed to look into the understanding and perception of antibiotics and AMR among community members in the Ashanti Region of Ghana. The aim is to explore current knowledge on the topic of antibiotics and AMR, whether AMR is perceived as a problem and if so, what possible solutions against AMR could look like. The aim is to gain insights into perspectives of different community members.

Interviews will be conducted with patients/customers exiting pharmacies and chemical shops in Agogo and the health centre in Ananekrom. The number of interviews will be determined by the reaching of saturation. The results will form part of the ground research to develop an AMR intervention adapted to the local context and needs.

**Objectives:**

- To gain insights into community members’ understanding and perception of antibiotics and AMR.
- To identify possible needs and challenges related to antibiotics and AMR.
- To identify possible solutions/interventions on AMR.

1. **Introduction**

- Introduction of interviewer.
- Explanation of project and interview procedure (participation voluntarily, not paid).
- Signing of informed consent form.

1. **Introduction questions**

- You have just excited the …. Did you get a prescription or antibiotics?
- If not, do you still remember the last time you used antibiotics?

1. **. Antibiotics**

- Do you know what antibiotics are and how would you describe them?

*[If the interviewee does not know what antibiotics are show them examples. Use toupaye/red-yellow pill]*

- According to you understanding, what is the purpose of antibiotics?
  - How would you describe the symptoms you have when you are taking antibiotics?
  - Can you know from the symptoms if you need antibiotics or not? Can the healthcare workers know? How do they know?
- Could you give me any examples of antibiotics you know or already used?
  - How do you identify whether a drug is an antibiotic? (name, packaging, colour)
  - Do you still remember what symptoms you experienced when you last used antibiotics?
- Do you still remember where you bought antibiotics when you last needed them?
  - Could you please describe the process of getting antibiotics?
  - Is it easy to get antibiotics? Where can you get them?
  - Could you describe any facilitators or barriers you might have experienced when last getting antibiotics?
  - Did it already happen to you that you wanted to have a specific medication, but the healthcare worker did not want to prescribe it to you?
- What is your experience with antibiotics? (positive/ negative, side effects, efficiency, instructions given)

1. **AMR**

- Have you heard about AMR already?

*[If the interviewee does not know AMR explain it using Damien’s cartoon]*

- - If yes, where and what?
  - How would you describe AMR?
- According to your opinion, what is causing and driving AMR?
  - Can you think of any practices that can lead to AMR?
    (human medicine, animal breeding, environment)
- How important do you think the topic of AMR is?
  - What are the dangers or problems related to AMR?
  - Does AMR have any consequences to you personally or your community? If so, which?
  - Does AMR have any consequences on animals or the environment? If so, which?
- According to your perception, is AMR discussed in Ghana? If yes, since when and how?
  - Do you think that AMR is national/regional/international problem? Why?

1. **AMR action**

- Have you heard or seen any campaign, pictures, radio messages, or other information about AMR? If so, what was the main message?
- What are do you think could be helpful to improve the situation regarding AMR?
  - Who should be involved in your suggested solutions?
  - Who should be targeted by such an intervention? (stakeholders, sectors)
- What role could you play in possible future action related to AMR?
- What role could your community play in possible future action related to AMR?

1. **End/conclusion of interview**

- Thank for time and valuable answers. Outlook.
