# Supplementary material for: Exploring the Current Situation and Developing Strategies for Behavior Change to Improve Antibiotic Use in West Africa: Protocol for a Multidisciplinary Interventional Research Project
Source: JMIR Res Protoc. 2025 Jul 25;14:e66424. doi: 10.2196/66424 (PMC12334893; doi:10.2196/66424)
Supplement: Multimedia Appendix 7 [file resprot_v14i1e66424_app7.docx]

**Phase 2. – interventional research component**

**Interview Guide (Institutional) Actors AMR – Human Health**

***Interview Procedure***

- Explain project and hand out flyer
- Introduce myself and explain my role in project 🡪 (1) understand viewpoints of actors with regards to AMR, (2) another project phase where actors will be involved in discussions about interventions

**1) Before recording**

- Profession, function, years of experience

**2) AMR – General**

- Perception and importance of AMR & determinants of AMR, importance of AMR
- Determinants and influences of AMR (e.g., WASH, SES)
- Causes and drivers of AMR (human/veterinary medicine, animal breeding, pharmaceutical industry)
- Consequences of AMR on work and health care sector
- AMR in Ghana: discussed since when, particular for Ghana/neighbouring countries
- Description of institution’s work and activities
- Specific training received on AMR

**3) AMR – Health care sector**

- Knowledge and awareness of AMR among general population
- Knowledge and awareness of AMR in health care sector
- Doctors, physician assistants, nurses, pharmacists, licensed drug sellers
- AMR part of education/trainings?
- How, where, from whom to get antibiotics
- Access vs. excess: where and how to get antibiotics 🡪 different levels (licensed over the counter, hospital pharmacy, etc.)
- Prescription process and regulations
- Quality of generic antibiotics? Counterfeit/substandard medicines
- Payment of antibiotics (NHIS)
- Healthcare facilities:
- IPC, antimicrobial stewardship program
- Diagnostic tools
- Surveillance of disease and AMU

**4) AMR – Action**

- Actors involved in AMR & actions taken on AMR: which and since when, effectiveness/obstacles, NAP
- Future action and needs, possible interventions 🡪 stakeholder groups to target
